# Assessing seromuscular layer and serosa removal on intestinal permeability measurements in weaned piglet everted sac segments

**DOI:** 10.1093/jas/skae148

**Published:** 2024-05-28

**Authors:** Lonneke Noorman, Bart van der Hee, Myrthe S Gilbert, Sonja de Vries, Sylvia van der Hoek, Walter J J Gerrits

**Affiliations:** Department of Population Health Sciences, Faculty of Veterinary Medicine, Utrecht University, 3584CL Utrecht, The Netherlands; Host-Microbe Interactomics, Department of Animal Sciences, Wageningen University & Research, 6700AH Wageningen, The Netherlands; Laboratory of Microbiology, Agrotechnology and Food Sciences, Wageningen University & Research, 6700EH Wageningen, The Netherlands; Animal Nutrition Group, Department of Animal Sciences, Wageningen University & Research, 6700AH Wageningen, The Netherlands; Animal Nutrition Group, Department of Animal Sciences, Wageningen University & Research, 6700AH Wageningen, The Netherlands; Department of Population Health Sciences, Faculty of Veterinary Medicine, Utrecht University, 3584CL Utrecht, The Netherlands; Animal Nutrition Group, Department of Animal Sciences, Wageningen University & Research, 6700AH Wageningen, The Netherlands

**Keywords:** absorption capacity, everted gut sac, intestinal barrier function, leaky gut, stripping intestinal segments, stripping the seromuscular layer and serosa

## Abstract

The integrity of the intestinal barrier is crucial for regulating the passage of pathogens and toxins, while facilitating nutrient absorption. The everted gut sac technique, an ex-vivo technique, can be used to study interventions on barrier function. This cost-effective approach utilizes relatively large gut segments to study specific intestinal regions. Typically, intact (non-stripped) intestinal segments are used, but their use may underestimate permeability due to the medial positioning of blood vessels relative to the seromuscular layer and serosa. However, removing these layers risks physical damage, resulting in an overestimation of intestinal permeability. Therefore, we investigated the impact of stripping jejunal segments on permeability to fluorescein isothiocyanate-dextran (FITC, 4 kDa) and tetramethylrhodamine isothiocyanate-dextran (TRITC, 40 kDa), and on the absorption of glucose, lysine, and methionine in jejunal segments from 80 piglets at 8 d postweaning. Piglets were subjected to either high or low sanitary housing conditions and diets provoking intestinal protein fermentation or not, expected to influence intestinal permeability. Stripping of the seromuscular layer and serosa increased the passage of 4 kDa FITC-dextran (stripped vs. non-stripped; 1.1 vs. 0.9 pmol/cm^2^/min, *P* < 0.001), glucose (40.0 vs. 19.1 pmol/cm^2^/min, *P* < 0.001), lysine (2.5 vs. 2.0 nmol/cm^2^/min, *P* < 0.001), and methionine (4.1 vs. 2.7 pmol/cm^2^/min, *P* < 0.001). As permeability increased, the differences in methionine passage between stripped and non-stripped intestinal segments also increased (slope = 1.30, *P* = 0.009). The coefficients of variation were comparable between stripped and non-stripped intestines (over all treatments, stripped vs. non-stripped 38% vs. 40%). Stripping, by isolating mucosal processes without introducing additional variation, is thus recommended for studies on intestinal permeability or absorption.

## Teaser

Stripping the seromuscular layer and serosa is preferred to study intestinal permeability or absorption using the everted gut sac technique. This method involves creating isolated, everted, intestinal sacs to measure the translocation of substances of interest into these sacs.

## Introduction

The semipermeable intestinal barrier selectively absorbs nutrients while restricting intraluminal pathogen and toxin translocation (Nusrat et al. 2000; Groschwitz and Hogan 2009). Permeability through this barrier is regulated by intramembrane proteins connecting the intestinal epithelium consisting of desmosomes, adherens junctions, and tight junctions. Desmosomes and adherens junctions play an important role in the strong adhesion between cells, while tight junctions mediate transport through the intercellular junctions (Groschwitz and Hogan 2009). Paracellular transport can be regulated by altering the expression of tight junctions and by modulating the tension within the actin-myosin ring (Nusrat et al. 2000; Bruewer and Nusrat 2006). Under healthy conditions, there is passive diffusion of water, electrolytes, and other small molecules (<600 Daltons) through the intercellular junctions (Watson et al. 2001). When the barrier function is compromised, larger molecules and antigens can translocate, exacerbating the risk of local and systemic inflammation.

The everted gut sac technique is a commonly used ex-vivo technique to study intestinal permeability (Acra and Ghishan 1991; Alam et al. 2011; Liu et al. 2016). The advantages of this method are that specific regions of the intestine can be studied and that relatively large gut segments can be used. Moreover, this method is relatively easy to perform and cost-effective (Deferme et al. 2008). Intact (non-stripped) intestinal segments are commonly used (Alam et al. 2011). However, the location of blood vessels medial to the seromuscular layer and serosa suggests that including these layers may lead to an underestimation of intestinal permeability and absorption (Wolfe et al. 1973). Therefore, it may be preferable to remove the seromuscular layer and serosa before analyzing intestinal permeability. Nevertheless, the process of stripping these layers is time-consuming and might increase the risk of inducing physical damage to the everted gut sac segment.

This experiment aimed to assess and compare jejunal permeability and active nutrient transport using both non-stripped and stripped jejunal segments in the everted gut sac technique. The pigs were subjected to either high or low sanitary housing conditions and diets high or low in protein, anticipated to influence intestinal permeability and absorption. Two markers of different sizes (FITC-dextran, 4 kDa, and TRITC-dextran, 40 kDa) were used to assess permeability, assuming that the large TRITC-dextran will only pass when the intestinal epithelium is considerably damaged, whereas the smaller FITC-dextran will also pass with minor epithelial damage. For active nutrient transport, the transport of a neutral (methionine) and basic (lysine) amino acid were measured, together with glucose, which uses different transporters (Bröer and Fairweather 2018; Chen et al. 2018; Noorman et al. 2023). Acidic amino acids were not included, because they mainly serve as an energy source for the intestines (Bröer and Fairweather 2018). We hypothesized that stripping the intestinal segments increases both intestinal permeability and active nutrient transport, but also potentially increases variation due to physical damage to the intestinal segment.

## Material and Methods

A project license was granted by the Central Committee for Animal Experimentation (AVD1,040,020,209,705, The Hague, the Netherlands) and the experiment was approved by the Animal Welfare Body of Wageningen University (2020.W-0012.001).

### Experimental design

This experiment was part of a larger study on the effects of high-protein diets on protein fermentation and intestinal health in weaned piglets kept under high and low sanitary housing conditions (Noorman et al. 2023). The experiment was designed in a 2 × 2 × 2 factorial arrangement, with stripped and non-stripped jejunal segments (S + and S−), low or high dietary indigestible protein contents (LiP and HiP), and high or low sanitary conditions (HSC and LSC). In total, 160 female piglets (TN70 × Tempo; Topigs, Helvoirt, The Netherlands) were selected from a commercial farm in the Netherlands, in five batches of 32 piglets each. Immediately after weaning (mean age ± SEM; 29 ± 0.3 d, mean body weight ± SEM; 9.1 ± 0.21 kg), piglets were transported to the research facilities of Wageningen University & Research.

The piglets were housed as described previously (Noorman et al. 2023). Briefly, piglets were divided over four climate-controlled respiration chambers with two pens (220 × 110 cm each) per chamber and four piglets per pen. In the HSC rooms, feces were removed per pen twice daily. In LSC, the feces were not removed, and in addition, feces collected from pens of weaned piglets (<25 kg) and sows on five commercial farms were spread on the floor twice a week to enhance antigenic pressure. The rooms of HSC and LSC were separated and each had its own entrance and barrier measures to maintain the contrast in hygiene.

For the first 3 d, heat lamps were provided and the temperature was controlled at 26 °C. Subsequently, the temperature was gradually adjusted to reach 28 °C, and the heat lamps were removed. The piglets were exposed to 16 h of light (08.00 to 24.00 hours). Relative humidity was maintained at 65%.

Upon arrival, piglets were allocated to eight pens, based on their initial body weight and litter origin, to minimize variation in body weight among pens and to exclude littermates in the same treatment. After one week, half of the piglets (out of two pairs, the pair with the highest feed intake was selected; Noorman et al. 2023) were euthanized and two jejunal segments (non-stripped and stripped) per pig were collected to study permeability and absorption capacity using the everted gut sac technique.

### Diets and feeding

After arrival, the piglets were allocated to the treatments and immediately switched to one of the experimental diets (LiP or HiP). Each diet was provided in four pens; two pens in LSC and two pens in HSC. The diets and feeding schedule are described by Noorman et al. (2023). Briefly, two diets were formulated containing either casein with high-protein digestibility (LiP), or sunflower seed meal with low digestibility (HiP), as the main protein source (CVB, 2018), creating a difference in indigestible protein (26 vs. 42 g/kg), thereby influencing the colonic protein influx. All diets met the nutritional requirements of weaned piglets (CVB, 2020). To habituate the piglets to the experimental diets, a 50:50 (w/w) mixture of both diets was introduced on the commercial farm, 2 d before transport to the research facilities.

### Everted sac technique

On day 8, half of the piglets were euthanized as described by Noorman et al. (2023). The small intestine was extracted, and a 40 cm segment at the midpoint of the small intestine (duodenum + jejunum + ileum) was cleared of intestinal contents through gentle squeezing. Two segments of 20 cm per pig were used to measure intestinal permeability and glucose-, lysine-, and methionine absorption with the everted gut sac technique (Wilson and Wiseman, 1954). In one of the two segments (randomly selected), the seromuscular layer and serosa were carefully removed manually. This stripping procedure was alternately performed by two trained persons. In the other segment, no layers were removed. Then, segments were flushed with phosphate-buffered saline and inverted, filled with Ringer solution (2.25 g/L NaCl, 0.105 g/L KCl, 0.06 g/L CaCl_2_, 0.05 g/L NaHCO_3_, and 2 mM HEPES) and closed by means of a ligature with a rubber band. An additional ligature was then placed in the center to divide the segments into two intestinal sacs to minimize sample loss in case of leakage. The sacs were then placed in an Erlenmeyer flask filled with 150 mL Ringer solution containing 2 mM HEPES, 5 mM glucose, 30 μg/mL fluorescein isothiocyanate (FITC)-dextran (4 kD), 30 μg/mL tetramethylrhodamine isothiocyanate (TRITC)-dextran (40 kD), 8 mM L-Lysine acetate and 8 mM l-Methionine, and incubated for 60 min at 39 °C. The Ringer solution was aerated before incubation by adding oxygen in the buffer via an air pump used for aquariums. After incubation, the sac content was collected and stored at −20 °C pending analyses, and the length and width of the flattened intestinal segment were measured to calculate the surface area. Sac contents were thawed at room temperature and analyzed for 4 kDa FITC-dextran and 40 kDa TRITC-dextran by a fluorometer (SpectraMax® M3 Multi-Mode Microplate Reader) and glucose was analyzed using a commercial assay kit (d-Glucose (glucose oxidase/peroxidase; GOPOD) Assay Kit; Megazyme, Wicklow, Ireland). Free lysine and methionine were measured in sac contents according to ISO 13,903:2005, where amino acids were separated by ion-exchange chromatography, and determined by post-column reaction with ninhydrin, using photometric detection at 570 nm.

### Calculations

The transport of 4 kDa FITC-dextran, 40 kDa TRITC-dextran, lysine, methionine, and glucose across the intestinal wall was calculated as follows:


$$\begin{array}{l} Transport\;(nmol\;or\;pmol/cm^{2}/\min)=\frac{Concentration\;\;\;intestinal\;\;\;sac\;volume\;\;\;added}{Mucosal\;\;\;surface\;\;\;area}\;/\;60, \end{array}$$

where concentration intestinal sac is the measured concentration of the compound of interest in nmol or pmol/mL, volume added is the amount of buffer added to the intestinal sac in mL, and mucosal surface area is the length of the intestinal segment (cm) × circumference (cm).

### Statistical analyses

For all statistical analyses, R for Windows 3.6.0 was used (packages: moments; Komsta and Novomestky 2022; ggplot2; Wickham 2016; and stats; R Core Team 2023).

Normality of the residuals of the response variables was checked graphically with quantile-quantile plots, and the Shapiro–Wilk test.

The relation between the transport of 4kDa FITC-Dextran, 40 kDa TRITC-dextran, glucose, lysine, and methionine across the intestinal wall measured in stripped and non-stripped intestinal segments was analyzed by simple linear regression, using the model *y = a + βx*; where *y* is the transport measured in stripped intestinal segments, *a* is intercept, *β* is slope and *×* is the transport measured in non-stripped intestinal segments. The difference between *y* and *x (y − x)* is the difference between the average transport in stripped vs. non-stripped jejunal segments, and the *P*-values indicate if *x = y* is rejected (*P* < 0.05 means that the hypothesis *x = y*, taken at *x = x̄*, was rejected). The *β* represents the slope of the regression line and the *P*-value indicates if the slope = 1 (*P* < 0.05 means that the hypothesis slope = 1 was rejected). The coefficient of determination (R^2^) was adjusted for the number of terms in the model. Results are described as means. The pig was considered as experimental unit.

## Results and Discussion

The passage of 4 kDa FITC-dextran through non-stripped jejunal segments varied between 0.4 and 2.3 pmol/cm^2^/min, corresponding to 0.2% and 0.8% of 4 kDa FITC-dextrans added to the buffer. Despite different methodologies used, this can be considered in the mid-range compared with studies in rats (Gao et al. 2001; Lambert et al. 2002, Qin and Deitch, 2015). The passage of glucose in stripped jejunal segments varied between 5.7 and 178.5 pmol/cm^2^/min, corresponding to 0.01% and 0.11% of glucose added to the buffer. The passage of lysine in stripped jejunal segments varied between 1.3 and 4.6 nmol/cm^2^/min, corresponding to 0.5% and 2.3% of lysine added to the buffer. The passage of methionine varied between 1.3 and 8.5 nmol/cm^2^/min, corresponding to 0.6% and 3.9% of methionine added to the buffer. Despite different methodologies used, the passage of glucose, lysine, and methionine can be considered low compared to studies in humans (Rey et al. 1974), mice (Hamilton and Butt 2013), and rats (Rider et al. 1967; Nolles et al. 2007; Ouassou et al. 2018). The intestinal passage of glucose, lysine, and methionine may be influenced by the metabolism in the enterocytes, where glucose, together with glutamine and glutamate, is the main energy source for the intestinal epithelial cells (Stoll et al. 1999; Chen et al. 2018; Guevarra et al. 2018). The dosage of glucose added to the buffer in our study (5 mM) was relatively low compared to the other studies (10 to 3330 mM), which might increase the relative contribution of glucose to enterocyte metabolism.

Treatment effects of sanitary conditions and diet on intestinal permeability were limited (Noorman et al. 2023). In stripped intestinal segments, only the HiP diet slightly increased the intestinal translocation of 4 kDa FITC-dextran and 40 kDa TRITC-dextran (4 kDa FITC-dextran, HiP vs. LiP; 1.15 vs. 1.06 pmol/cm^2^/min, and 40 kDa TRITC-dextran; 0.26 vs. 0.12 pmol/cm^2^/min). In this study, the treatment effects of sanitary conditions and diet were independent of stripping (interaction stripping × diet and stripping × sanitary conditions *P* > 0. 10), but interactions may be seen in studies using interventions with greater effects on intestinal permeability.

**Figure 1.**
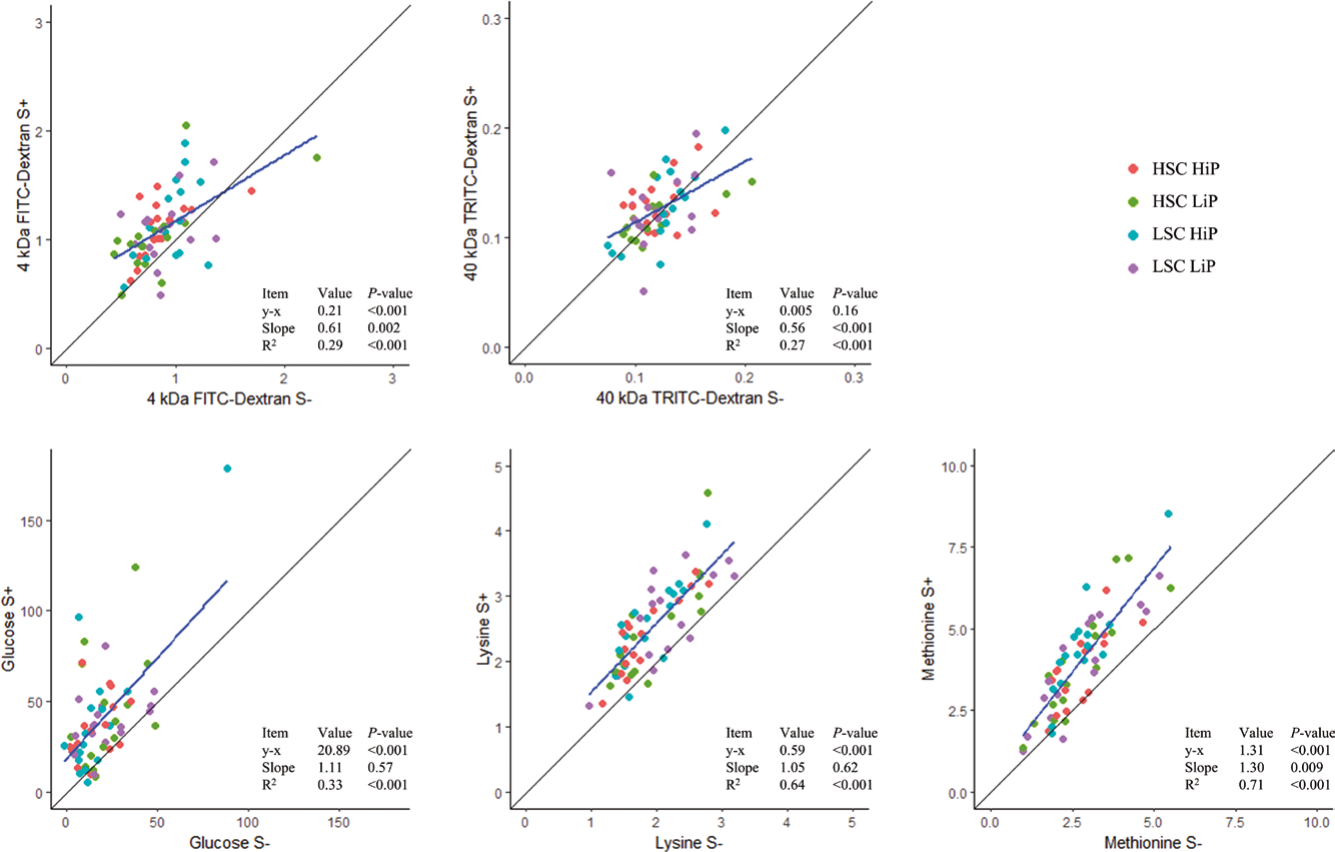
Linear regression analyses of intestinal permeability to 4 kDa FITC-dextran and 40 kDa TRITC-dextran (pmol/cm^2^/min) and transport of glucose (pmol/cm^2^/min), lysine (nmol/cm^2^/min), and methionine (nmol/cm^2^/min) across the jejunal wall in stripped and non-stripped jejunal segments of weaned piglets kept under high (HSC) or low (LSC) sanitary conditions and fed a diet low (LiP) or high (HiP) in indigestible proteins. The solid lines through the origin represent y = x. y − x is the difference between the average permeability in stripped vs. non-stripped jejunal segments and *P* < 0.05 means that the hypothesis x = y is rejected. The *P*-value of the slope indicates if the slope significantly differs from 1. R^2^ is adjusted for the number of terms in the model.

Stripping of the seromuscular layer and serosa increased the passage of 4kDa FITC-dextran (stripped vs. non-stripped; 1.1 vs. 0.9 pmol/cm^2^/min, *P* < 0.001, Figure 1), glucose (40.0 vs. 19.1 pmol/cm^2^/min, *P* < 0.001), lysine (2.5 vs. 2.0 nmol/cm^2^/min, *P* < 0.001), and methionine (4.1 vs. 2.7 nmol/cm^2^/min, *P* < 0.001). These findings align with the observations of Wolfe et al. (1973), who reported a 1.5- to 1.8-fold higher absorption rate of salicylate in stripped intestinal segments of rats compared to non-stripped counterparts. Glucose, lysine, and methionine are transported through the mucosa via active transport mechanisms facilitated by different transporters, whereas 4 kDa FITC-dextran is transported by paracellular transport (Bröer and Fairweather 2018; Chen et al. 2018). The presence of the seromuscular layer and serosa in non-stripped intestinal segments may serve as an additional barrier, impeding the passage of markers and nutrients. The positioning of blood vessels medial to the seromuscular layer and serosa in vivo suggests that the use of stripped intestinal segments more accurately reflects mucosal permeability. No differences in the passage of 40 kDa TRITC-dextran were found between stripped and non-stripped intestinal segments (*P* = 0.16).

To study intestinal paracellular transport, usually, markers of comparable size to 4 kDa FITC-dextran or smaller are used (Wolfe et al. 1973; Gao et al. 2001; Lambert et al. 2002). It may be that 40 kDa TRITC-dextran, due to its relatively large size, was transported via transcytosis and basolateral exocytosis rather than via paracellular transport (Maiti 2017; Meijers et al. 2018; Rousou et al. 2022), presumably not affected by stripping.

Whether the effect of stripping depends on the degree of permeability, can be assessed based on the slope of the regression line (**Figure 1**). However, using stripped or non-stripped intestinal segments as a dependent variable potentially influences the slope, and thus the conclusion. The regression line is calculated by ∆ y/∆x. For 4 kDa FITC-dextran and 40 kDa TRITC-dextran, for example, the differences between stripped and non-stripped intestinal segments decreased as permeability increased in the current analyses (slope ≠ 1, 4 kDa FITC-dextran *P* = 0.002, 40 kDa TRITC-dextran *P* < 0.001, **Figure 1**; stripped intestinal segments as dependent variable). In contrast, when non-stripped intestinal segments were used as the dependent variable, differences between stripped and non-stripped intestinal segments increased as permeability increased (**Supplementary Figure S1**). For glucose and lysine, the regression line in **Figure 1** was parallel to x = y (slope = 1), indicating that the effect of stripping is independent of the degree of permeability. However, when stripped intestinal segments were used as the dependent variable, the differences between stripped and non-stripped intestinal segments increased with increasing permeability (**Supplementary Figure S1**). Based on these contrasting results, we conclude no permeability-dependent response was demonstrated for 4kDa FITC-dextran, 40 kDa TRITC-dextran, glucose, and lysine. For methionine, the differences between stripped and non-stripped intestinal segments increased with increased permeability (slope ≠ 1, *P* = 0.009, **Figure 1**). This trend was consistent regardless of whether stripped or non-stripped intestinal segments were used as dependent variable.

The seromuscular layer and serosa were stripped by careful manual removal. In the majority of intestinal segments, the seromuscular layer and serosa were successfully removed as a single intact layer. Nevertheless, variations in layer thickness were observed, making the removal process more challenging in certain samples. Consequently, these instances required additional time and manipulation, potentially leading to unintentional physical damage to the everted gut sac segment. Furthermore, the stripping procedure may have been influenced by the individual performing it, but collective training in layer removal minimized these effects as much as possible. In this study, no histological imaging was performed to determine whether any damage had occurred. However, the coefficient of variation was similar in non-stripped and stripped intestines for the transport of 4 kDa FITC-dextran (stripped vs. non-stripped 30% vs. 34%), 40 kDa TRITC-dextran (22% vs. 22%), lysine (27% vs. 27%), methionine (39% vs. 37%), and glucose (72% vs. 79%), indicating that stripping did not reduce the precision of the assay. This is in agreement with the results of histological imaging performed in the study of Neirinckx et al. (2010) and Wolfe et al. (1973), in which the procedure of removing the seromuscular layer and serosa from intestinal segments in pigs and rats did not substantially alter the integrity of the epithelium, lamina propria, or submucosa.

In this study, stripping was performed on segments of the small intestine. No comparison has been made between stripped and non-stripped segments of the large intestine, but we experienced that, although more challenging, stripping is also possible in the large intestine (Noorman et al. 2023).

## Conclusions

Using stripped intestinal segments resulted in increased permeability to small molecules (<4kDa) and enhanced absorption of lysine, methionine, and glucose. A permeability-dependent response was specifically identified for methionine. Notably, the variation in non-stripped and stripped intestinal segments remained comparable, affirming that stripping did not compromise assay precision. Given that stripping aligns more closely with the physiological conditions by not passing the serosa and seromuscular layer, while maintaining assay precision, the removal of the seromuscular layer and serosa is recommended to evaluate absorption and permeability using the everted gut sac technique.

## Supplementary Material

skae148_suppl_Supplementary_Figure_S1

## Abbreviations

FITC: fluorescein isothiocyanate-dextran
TRITC: tetramethylrhodamine isothiocyanate-dextran

## References

[CIT0001] Acra, S. A., and F. K. Ghishan. 1991. Methods of investigating intestinal transport. JPEN J. Parenter. Enteral. Nutr. 15:93S–98S. doi: 10.1177/014860719101500393S 1865567

[CIT0002] Alam, M. A., F. I. Al-Jenoobi, and A. M. Al-mohizea. 2011. Everted gut sac model as a tool in pharmaceutical research: limitations and applications. J. Pharm. Pharmacol. 64:326–336. doi: 10.1111/j.2042-7158.2011.01391.x 22309264

[CIT0003] Bröer, S., and S. J. Fairweather. 2018. Amino acid transport across the mammalian intestine. Compr. Physiol. 9:343–373. doi: 10.1002/cphy.c170041 30549024

[CIT0004] Bruewer, M., A. Nusrat. 2006. Regulation of paracellular transport across tight junctions by the actin cytoskeleton. In: L. Gonzalez-Mariscal, ed, Landes Bioscience. Austin (TX): Springer Science + Business Media, LCC; p. 135–145.

[CIT0005] Central Bureau for Livestock Feeding (CVB), 2018. Chemical compositions and nutritional values of feed ingredients. The Netherlands, Lelystad: CVB.

[CIT0006] Central Bureau for Livestock Feeding (CVB), 2020. Voedernormen varkens en voederwaarden voedermiddelen voor varkens. The Netherlands, Lelystad: CVB.

[CIT0007] Chen, C., Y. Yin, Q. Tu, and H. Yang. 2018. Glucose and amino acid in enterocyte: absorption, metabolism and maturation. Front. Biosci. 23:1721–1739. doi: 10.2741/4669 29293459

[CIT0008] Deferme S. , P. Annaert, and P. Augustijns. 2008. In vitro screening models to assess intestinal drug absorption and metabolism. In: C. Ehrhardt, K-J. Kim, eds, Drug absorption studies, in situ, in vitro and in silico models. New York (NY): American association of pharmaceutical scientists; p. 182–215.

[CIT0009] Gao, F., M. Nakamaru, Y. Masubuchi, and T. Horie. 2001. Protective effect of a synthetic analog of prostaglandin E_1_ on the small intestinal damage induced by the administration of methotrexate to rats. J. Pharm. Sci. 90:1040–1048. doi: 10.1002/jps.1057 11536208

[CIT0011] Groschwitz, K. R., and S. P. Hogan. 2009. Intestinal barrier function: molecular regulation and disease pathogenesis. J. Allergy Clin. Immunol. 124:3–20; quiz 21. doi: 10.1016/j.jaci.2009.05.038 19560575 PMC4266989

[CIT0010] Guevarra, R. B., S. H. Hong, J. H. Cho, B. -R. Kim, J. Shin, J. H. Lee, B. N. Kang, Y. H. Kim, S. Wattanaphansak, R. E. Isaacson, et al. 2018. The dynamics of the piglet gut microbiome during the weaning transition in association with health and nutrition. J. Anim. Sci. Biotechnol. 9:54. doi: 10.1186/s40104-018-0269-6 30069307 PMC6065057

[CIT0012] Hamilton, K. L., and A. G. Butt. 2013. Glucose transport into everted sacs of small intestine of mice. Adv. Physiol. Educ. 37:415–426. doi: 10.1152/advan.00017.2013 24292921

[CIT0013] Komsta, L., Novomestky, F. 2022. _moments: Moments, cumulants, skewness, kurtosis and related tests_. R package version 0.13.1, https://CRAN.R-project.org/package=moments

[CIT0014] Lambert, G. P., C. V. Gisolfi, D. J. Berg, P. L. Moseley, L. W. Oberley, and K. C. Kregel. 2002. Molecular biology of thermoregulation, selected contribution: hyperthermia-induced intestinal permeability and the role of oxidative and nitrosative stress. J. Appl. Physiol. 92:1750–1761. doi: 10.1152/japplphysiol.00787.2001 11896046

[CIT0015] Liu, W., H. Pan, C. Zhang, L. Zhao, R. Zhao, Y. Zhu, and W. Pan. 2016. Developments in methods for measuring the intestinal absorption of nanoparticle-bound drugs. Int. J. Mol. Sci. 17:1171. doi: 10.3390/ijms17071171 27455239 PMC4964542

[CIT0016] Maiti, S. 2017. Nanometric biopolymer devices for oral delivery of macromolecules with clinical significance. In: A. M. Grumezescu, ed, Multifunctional systems for combined delivery, biosensing and diagnostics. Amsterdam: Elsevier; p. 109–138.

[CIT0017] Meijers, B., R. Farré, S. Dejongh, M. Vicaro, and P. Evenepoel. 2018. Intestinal barrier function in chronic kidney disease. Toxins. 10:298. doi: 10.3390/toxins10070298 30029474 PMC6071212

[CIT0018] Neirinckx, E., C. Vervaet, J. Michiels, S. de Smet, W. van den Broeck, J. P. Remon, P. De Backer, and S. Croubels. 2010. Feasibility of the ussing chamber technique for the determination of *in vitro* jejunal permeability of passively absorbed compounds in different animal species. J. vet. Pharmacol. Therap. 34:290–297. doi: 10.1111/j.1365-2885.2010.01218.x 21492193

[CIT0019] Nolles, J. A., I. G. S. Peeters, B. I. Bremer, R. Moorman, R. E. Koopmanschap, M. W. A. Verstegen, and V. V. A. M. Schreurs. 2007. Dietary amino acids fed in free form or as protein do differently affect amino acid absorption in a rat everted sac model. K. Anim. Physiol. Anim. Nutr. 92:529–537. doi: 10.1111/j.1439-0396.2007.00743.x 19012596

[CIT0020] Noorman, L., M. S. Gilbert, B. van der Hee, S. de Vries, and W. J. J. Gerrits. 2023. Low sanitary housing conditions increase protein fermentation in piglets but do not aggravate the effects of protein fermentation on intestinal health. Anim. Feed Sci. Technol. 301:115669. doi: 10.1016/j.anifeedsci.2023.115669

[CIT0021] Nusrat, A., J. R. Turner, and J. L. Madara. 2000. Molecular physiology and pathophysiology of tight junctions IV. Regulation of tight junctions by extracellular stimuli: nutrients, cytokines, and immune cells. Am. J. Physiol. Gastrointest. Liver Physiol. 279:G851–G857. doi: 10.1152/ajpgi.2000.279.5.G851 11052980

[CIT0022] Ouassou, H., T. Zahidi, S. Bouknana, M. Bouhrim, H. Mekhfi, A. Ziyyat, A. Legssyer, M. Aziz, and M. Bnouham. 2018. Inhibition of α-glucosidase, intestinal glucose absorption, and antidiabetic properties by Caralluma europaea. eCAM. 2018:9589472. doi: 10.1155/2018/9589472 30228829 PMC6136516

[CIT0023] Qin, X., and E. A. Deitch. 2015. Dissolution of lipids from mucus: a possible mechanism for prompt disruption of gut barrier function by alcohol. Toxicol. Lett. 232:356–362. doi: 10.1016/j.toxlet.2014.11.027 25445722 PMC4291284

[CIT0024] R Core Team. 2023. R: A language and environment for statistical computing. R foundation for statistical computing, Vienna, Austria. https://www.R-project.org/

[CIT0025] Rey, F., F. Drillet, J. Schmitz, and J. Rey. 1974. Influence of flow rate on the kinetics of the intestinal absorption of glucose and lysine in children. Gastroenterology 66:79–85. doi: 10.1016/S0016-5085(74)80082-X 4809503

[CIT0026] Rider, A. K., H. P. Schedl, G. Nokes, and S. Shining. 1967. Small intestinal glucose transport, proximal-distal kinetic gradients. J. Gen. Physiol. 50:1173–1182. doi:10.1085/jgp.50.5.1173 6033580 PMC2225704

[CIT0027] Rousou, C., J. de Maar, B. Qiu, K. van der Wurff-Jacobs, M. Ruponen, A. Urtti, S. Oliveira, C. Moonen, G. Storm, E. Mastrobattista, et al. 2022. The effect of micro-bubble-assisted ultrasound on molecular permeability across cell barriers. Pharmaceutics. 14:494. doi: 10.3390/pharmaceutics14030494 35335871 PMC8949944

[CIT0028] Stoll, B., D. G. Burrin, J. Henry, H. Yu, F. Jahoor, and P. J. Reeds. 1999. Substrate oxidation by the portal drained viscera of fed piglets. Am. J. Physiol. 277:E168–E175. doi: 10.1152/ajpendo.1999.277.1.E168 10409141

[CIT0029] Watson, C. J., M. Rowland, and G. Warhurst. 2001. Functional modeling of tight junctions in intestinal cell monolayers using polyethylene glycol oligomers. Am. J. Physiol. Cell Physiol. 281:C388–C397. doi: 10.1152/ajpcell.2001.281.2.C388 11443038

[CIT0030] Wickham, H. 2016. ggplot2: Elegant Graphics for Data Analysis. New York: Springer-Verlag.

[CIT0031] Wilson, T. H., and G. Wiseman. 1954. The use of sacs of everted small intestine for the study of the transference of substances from the mucosal to the serosal surface. J. Physiol. 123:116–125. doi: 10.1113/jphysiol.1954.sp005036 13131249 PMC1366157

[CIT0032] Wolfe, D. L., S. C. Forland, and L. Z. Benet. 1973. Drug transfer across intact rat intestinal mucosa following surgical removal of serosa and muscularis externa. J. Pharm. Sci. 62:200–205. doi: 10.1002/jps.2600620203 4686389

