# Supplementary material for: Assessing seromuscular layer and serosa removal on intestinal permeability measurements in weaned piglet everted sac segments
Source: J Anim Sci. 2024 May 28;102:skae148. doi: 10.1093/jas/skae148 (PMC11222984; doi:10.1093/jas/skae148)
Supplement: skae148_suppl_Supplementary_Figure_S1 [file skae148_suppl_supplementary_figure_s1.pdf]

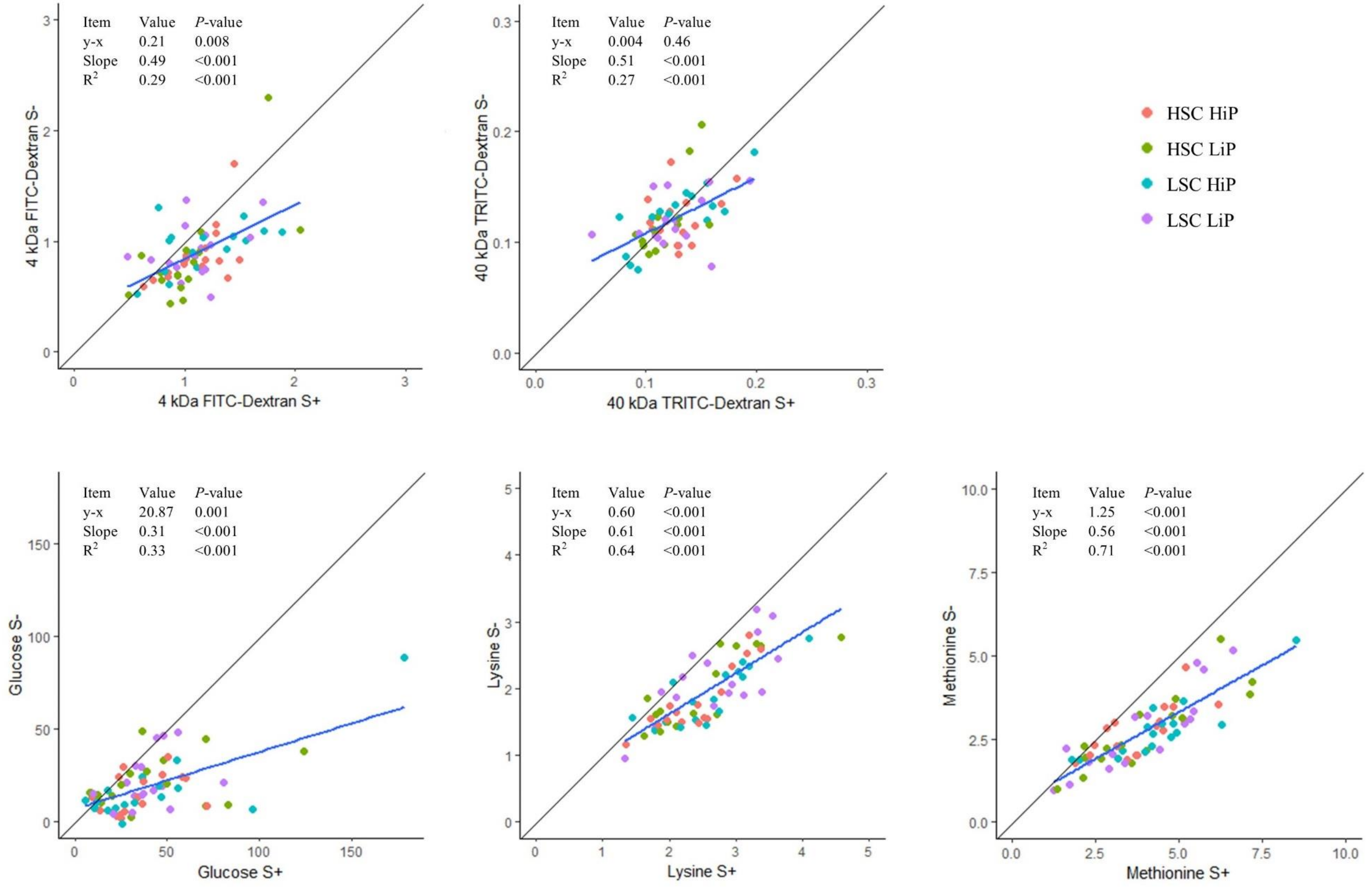

Figure S1: Linear regression analyses of intestinal permeability to 4 kDa FITC-dextran and 40 kDa TRITC-dextran (pmol/cm^2/min) and transport of glucose (pmol/cm^2/min), lysine (nmol/cm^2/min), and methionine (nmol/cm^2/min) across the jejunal wall in stripped and non-stripped jejunal segments of weaned piglets kept under high (HSC) or low (LSC) sanitary conditions and fed a diet low (LiP) or high (HiP) in indigestible proteins, with intact intestinal segments as dependent variable. Black lines represent y=x. y−x is the difference between the average permeability in stripped vs. non-stripped jejunal segments and $P<0.05$ means that the hypothesis x=y is rejected. The *P*-value of the slope indicates if the slope significantly differs from 1. R^2 is adjusted for the number of terms in the model.
